# Reevaluation of Physical and Chemical Singlet Oxygen Quenching Efficiency of Antioxidants via a Specific Methanol-Soluble Trap

**DOI:** 10.3390/antiox15050625

**Published:** 2026-05-14

**Authors:** Dongkun Zhao, Xin Wang, Lijuan Wang, Qing Ma, Jingwen Li, Baocheng Xu, Xinjing Dou, Lili Liu

**Affiliations:** 1 College of Food and Bioengineering, Henan University of Science and Technology, Luoyang 471023, China; kun18238796961@163.com (D.Z.); wangxin_327@163.com (X.W.); m15137666063@163.com (Q.M.); jingwen2268928436@163.com (J.L.); xinjingdou@haust.edu.cn (X.D.); yangliuyilang@126.com (L.L.); 2 College of Basic Medical Science, Ningxia Medical University, Yinchuan 750004, China; lijuan.wang@nxmu.edu.cn; 3 Henan International Joint Laboratory of Food Green Processing and Safety Control, Henan University of Science and Technology, Luoyang 471023, China; 4 National Experimental Teaching Demonstration Center of Food Processing and Safety, Henan University of Science and Technology, Luoyang 471023, China

**Keywords:** antioxidants, furfuryl alcohol, photooxidation, singlet oxygen, quenching

## Abstract

Furfuryl alcohol (FFA), a methanol-soluble and highly specific compound for singlet oxygen (^1^O_2_), was used as a ^1^O_2_ trapping agent to reevaluate physical and chemical ^1^O_2_ quenching efficiency of five antioxidants, namely α-tocopherol, butylated hydroxyanisole (BHA), tert-butylhydroquinone (TBHQ), epigallocatechin gallate (EGCG), and quercetin. The total ^1^O_2_ quenching rate constants of α-tocopherol, BHA, TBHQ, EGCG, and quercetin were 2.0 (±0.1) × 10^9^, 1.1 (±0.1) × 10^8^, 5.6 (±0.3) × 10^8^, 4.1 (±0.1) × 10^8^, and 2.2 (±0.1) × 10^8^ M^−1^s^−1^, respectively. The chemical quenching rate constants of α-tocopherol, BHA, TBHQ, EGCG, and quercetin were 1.4 × 10^7^, 2.0 × 10^6^, 7.7 × 10^6^, 2.0 × 10^7^, and 7.9 × 10^6^ M^−1^s^−1^, respectively. The percentages of chemical quenching in total ^1^O_2_ quenching were 0.7%, 1.8%, 1.4%, 4.8%, and 3.6% for α-tocopherol, BHA, TBHQ, EGCG, and quercetin, respectively, indicating that the five antioxidants quenched ^1^O_2_ almost exclusively by a physical quenching mechanism. This is the first report on the ^1^O_2_ quenching mechanism of quercetin in methanol solvent. The results of this study will provide theoretical guidance for the application of antioxidants to inhibit edible oil or fat-containing food from photooxidation.

## 1. Introduction

In the presence of oxygen, the natural pigments in vegetable oil, such as chlorophyll, are excited under light exposure and can generate reactive oxygen species via two distinct photosensitized oxidation pathways: Type I (secondary pathway) and Type II (main pathway) [1]. In the Type I pathway, the excited photosensitizer transfers an electron or hydrogen atom to a substrate or molecular oxygen, producing free radicals such as superoxide anion (O_2_^−^•) and hydroxyl radical (•OH). In the Type II pathway, energy is transferred from the excited photosensitizer to ground-state molecular oxygen (^3^O_2_), generating highly reactive singlet oxygen (^1^O_2_) [2]. Once ^1^O_2_ is generated, it reacts directly with the double bonds in unsaturated fatty acids via a concerted “ene” addition mechanism to generate hydroperoxide, which is further decomposed in secondary oxidations. Due to its high energy and reactivity, ^1^O_2_-induced oxidation of oils occurs at a rate over 1500 times faster than that caused by auto-oxidation [3,4]. Furthermore, many free radicals are produced in the process of photooxidation, which can further initiate automatic chain oxidation and increase the complexity of oxidation, resulting in rapid oxidation and deterioration of oil and fats and loss of nutritional value [5,6]. Therefore, inhibiting the formation of ^1^O_2_ in oil and fats is crucial for protecting them from oxidative degradation.

To inhibit the formation of ^1^O_2_ or quench ^1^O_2_ in an oil system, the most common method is to add a ^1^O_2_ quenching agent to the oil. In general, ^1^O_2_ quenching agents include natural antioxidants and synthetic antioxidants; natural antioxidants include tocopherols [7,8], carotenoids [9], flavonoids [10,11], etc. Synthetic antioxidants include tert-butylhydroquinone (TBHQ), tert-butylhydroxyanisol (BHA), tert-di-butylhydroxytoluene (BHT), and others [12,13]. In terms of mechanism, ^1^O_2_ quenching methods include physical quenching and chemical quenching. Chemical quenching means that the reaction between ^1^O_2_ and an antioxidant not only leads to the deactivation of ^1^O_2_ but also results in a rapid consumption of antioxidants and the formation new compounds, so its antioxidant life is short [14]. In contrast, in physical quenching, ^1^O_2_ is converted into ^3^O_2_ by either energy transfer or charge transfer, and there is no degradation of the antioxidant [15]. To select and obtain a high-efficiency ^1^O_2_ antioxidant, it is necessary to measure its physical quenching and chemical quenching rate constants, a popular topic in the field of oil antioxidation research. At present, studies on the ^1^O_2_ quenching rate constant of common antioxidants have been performed mainly in methanol systems [16,17], ethanol systems [18,19,20], pyridine systems [21], and methylene dichloride systems [22]. In a classical singlet oxygen quenching experiment, Foote et al. [23] employed 2,5-diphenyl-3,5-benzofuran (DPBF) as a probe and generated ^1^O_2_ photochemically using methylene blue (MB). The total and chemical ^1^O_2_ quenching rate constants for α-tocopherol were determined as 6.7 × 10^8^ M^−1^s^−1^ and 4.6 × 10^7^ M^−1^s^−1^ in methanol, respectively, by applying a steady-state kinetic equation. Similarly, Kim et al. [16] used DPBF as a probe in a methanol system, with ^1^O_2_ generated through the chemical reaction of NaMoO_4_ and H_2_O_2_. By this method, the total ^1^O_2_ quenching rate constants determined for BHA, BHT, TBHQ, and α-tocopherol were 3.37 × 10^7^, 4.26 × 10^6^, 1.67 × 10^8^, and 3.54 × 10^8^ M^−1^s^−1^, respectively. Additionally, an alternative ^1^O_2_ trapping agent has been reported for the determination of the ^1^O_2_ quenching constants of BHA, TBHQ, and BHT in methanol. Lee and Jung [17] used α-terpinene as a probe and MB as a photosensitizer generating ^1^O_2_ photochemically. Their experiments yielded total ^1^O_2_ quenching rate constants of 5.14 × 10^7^, 1.99 × 10^8^, and 3.41 × 10^6^ M^−1^s^−1^ for BHA, TBHQ, and BHT, respectively, with corresponding chemical ^1^O_2_ quenching rate constants of 3.90 × 10^5^, 2.93 × 10^6^, and 1.23 × 10^5^ M^−1^s^−1^. Beyond BHA, BHT, and TBHQ, catechins—natural compounds extracted from tea—are commonly employed as ^1^O_2_ quenching agents. Their total and chemical ^1^O_2_ quenching rate constants have been determined in a methanol system using similar methodologies. Specifically, the total ^1^O_2_ quenching rate constants for epicatechin gallate (ECG), epicatechin (EC), catechin (C), gallocatechin gallate (GCG), epigallocatechin gallate (EGCG), and epigallocatechin (EGC) were 4.83 × 10^7^, 2.99 × 10^7^, 1.66 × 10^7^, 1.09 × 10^8^, 1.31 × 10^8^, and 1.32 × 10^8^ M^−1^s^−1^, respectively [24]. Their chemical ^1^O_2_ quenching rate constants were 8.66 × 10^5^, 5.60 × 10^5^, 3.5 × 10^5^, 1.30 × 10^6^, 1.57 × 10^6^, and 1.63 × 10^6^ M^−1^s^−1^ for ECG, EC, C, EGC, GCG, and EGCG, respectively [24]. For quercetin (a natural antioxidant belonging to the hydroxyflavones family), its total ^1^O_2_ quenching rate constant was determined in ethanol [18,19] and methylene chloride systems [25]. Among these studies, Nagaoka et al. [18] investigated the solvent effect on the ^1^O_2_ quenching activity of hydroxyflavones using rose bengal to generate ^1^O_2_. They reported a total ^1^O_2_ quenching rate constant of 1.9 × 10^6^ M^−1^s^−1^ for quercetin in ethanol solution, determined via a near-infrared fluorescence lifetime measurement system. Notably, the total ^1^O_2_ quenching rate constant increased monotonically with solvent polarity (achieved by adding water to ethanol in the range of 0–25% *v*/*v*). Sibuea et al. [25] further studied quercetin’s effects on erythrosine-photosensitized oxidation of linoleic acid and palm oil in methylene chloride. Through a steady-state kinetic method, they estimated quercetin’s total ^1^O_2_ quenching rate constants as 3.2 × 10^9^ and 4.3 × 10^9^ M^−1^s^−1^ for linoleic acid and palm oil, respectively. However, to the best of our knowledge, the total and chemical ^1^O_2_ quenching rate constants of quercetin in methanol remain unreported.

In order to study the mechanism by which antioxidants quench ^1^O_2_, it is necessary to conduct real-time detection of ^1^O_2_ in the reaction system. At present, the commonly used methods are the direct detection method and the indirect detection method. Singlet oxygen can be detected directly via its characteristic near-infrared (NIR) phosphorescence emission at approximately 1270 nm [26]. Direct NIR phosphorescence detection offers high specificity and enables real-time, time-resolved measurements of ^1^O_2_ lifetime and quenching dynamics. However, this approach requires sophisticated instrumentation (e.g., sensitive NIR detectors) and is often limited by the low phosphorescence quantum yield of ^1^O_2_, particularly in polar solvents such as methanol [27]. In contrast, the indirect chemical trapping method using a ^1^O_2_ trapping agent is simple, cost-effective, and well-suited for steady-state kinetic studies in homogeneous solutions.

Previous studies have shown that determinations of the quenching rate constants of antioxidants have been carried out mostly in methanol systems and the probe used frequently is α-terpinene. However, the physical properties of α-terpinene show that it is hardly soluble in methanol and, according to the methods reported by Mukai et al. [20] and Kim et al. [16], this may lead to lower values for the total quenching rate constant of the antioxidants than the real ^1^O_2_ quenching rate. Another study used DPBF as a probe to determine the ^1^O_2_ quenching rate of TBHQ, BHT, and BHA, but some reports have shown that DPBF also reacts with superoxide anions, reducing the specificity of DPBF for ^1^O_2_ [28], which may influence the accuracy of the ^1^O_2_ quenching rates of the antioxidants. Therefore, selection of an ideal ^1^O_2_ probe for determining the quenching rate constant of an antioxidant is crucial and must meet the following requirements: (1) it must be soluble in the experimental system; (2) it must react specifically with ^1^O_2_ with a high rate constant; and (3) it should not physically quench ^1^O_2_. Furfuryl alcohol (FFA), being soluble in methanol and fulfilling all the aforementioned criteria, has been employed as a highly efficient probe for ^1^O_2_ in methanol solutions [29]. In our previous study, we established a simple, sensitive, and accurate high-performance liquid chromatography-UV (HPLC-UV) method for the quantitation of FFA [30]. This method facilitates the measurement of both total ^1^O_2_ quenching rate and chemical ^1^O_2_ quenching rate constants when using FFA as the ^1^O_2_ trapping agent.

In this study, FFA, a methanol-soluble and highly specific ^1^O_2_ reaction compound, was selected as a probe and MB was selected as a photosensitizer for ^1^O_2_-induced photooxidation. The goals of this study were as follows: (1) to investigate the relative protective activity of various antioxidants—α-tocopherol, BHA, TBHQ, EGCG, and quercetin—against ^1^O_2_-induced photooxidation of FFA in methanol; (2) to measure the total ^1^O_2_ quenching rate constants (*k_r_* + *k_q_*) and the chemical quenching rate constants (*k_r_*) of the antioxidants in methanol; and (3) to elucidate the quenching mechanisms of these antioxidants, with a particular focus on quercetin, whose quenching mechanism has not been previously reported. The evaluation of physical and chemical ^1^O_2_ quenching efficiency of the antioxidants will provide theoretical guidance for their application in protecting edible oil or fat-containing food from photooxidation.

## 2. Materials and Methods

### 2.1. Experimental Materials

Methylene blue (MB, purity ≥ 96%), tert-butylhydroquinone (TBHQ, purity ≥ 98%), butylated hydroxyanisole (BHA, purity ≥ 98%), and epigallocatechin (EGCG, purity ≥ 98%) were purchased from Aladdin Biochemical Co., Ltd., Shanghai, China. The α-tocopherol (purity ≥ 96%) and furfuryl alcohol (FFA, purity ≥ 98%) were purchased from Sigma Aldrich Co., Ltd. (St. Louis, MO, USA). Quercetin (purity ≥ 95%) was purchased from Shanghai Yuanye Biological Co., Ltd. (Shanghai, China). Methanol, formic acid, and acetonitrile (all chromatographically pure) were purchased from Sinopharm Chemical Reagent Co., Ltd. (Shanghai, China). Water was obtained from a MilliQ purification system (Millipore Corp., Burlington, MA, USA).

### 2.2. Standard Solutions Preparation

Seven stock standard solutions of MB, FFA, α-tocopherol, BHA, TBHQ, EGCG, and quercetin were prepared by dissolving a known amount of each compound into HPLC-grade methanol to a concentration of MB at 6.25 × 10^−5^ M, and FFA, α-tocopherol, BHA, TBHQ, EGCG, and quercetin at 1.0 × 10^−3^ M. Working solutions were obtained by successive dilution of the stock solutions.

### 2.3. Experimental Methods

#### 2.3.1. Determination of the Total ^1^O_2_ Quenching Rate Constant

The ^1^O_2_ quenching rate constant of α-tocopherol, BHA, TBHQ, EGCG, and quercetin in methanol was determined by using the reported method with a slight modification [16]. In our study, FFA was selected as a ^1^O_2_ probe, and ^1^O_2_ was generated through a Type II photochemical pathway using MB (which has an absorption maximum at ~660 nm) as the photosensitizer. The series sample solutions were prepared by dissolving a certain amount of FFA (as a probe, 2 × 10^−4^ M), antioxidants (including α-tocopherol, BHA, TBHQ, EGCG, and quercetin, 0 to 2 × 10^−3^ M), and MB (6.25 × 10^−5^ M) in methanol. Then, 10 mL of each sample solution was absorbed and added to a clean and transparent glass vial, and sealed with a screw cap. The prepared sample vials were transferred into an LED illumination box and exposed to 2000 lux of LED light for 25 min. Aliquots of sample solutions were collected at every 5-min interval to evaluate the effects of antioxidants on the FFA oxidation. The remaining content of FFA was determined by HPLC analysis. The ^1^O_2_ quenching rates for each antioxidant were carried out in triplet analysis.

#### 2.3.2. Determination of the Reaction Rate Constant Between FFA and ^1^O_2_

The chemical reaction rate constant between ^1^O_2_ and FFA in methanol was determined in this study. The series sample solutions were prepared by dissolving various contents of FFA (1~6 × 10^−4^ M) and MB (6.25 × 10^−5^ M) in methanol. Then, 10 mL of each sample solution was absorbed and added to a clean and transparent glass vial, and sealed with a screw cap. The prepared sample vials were transferred into an LED illumination box and exposed to 2000 lux of LED light for 25 min. Aliquots of sample solutions were collected at every 5-min interval to monitor the remaining content of FFA in the solutions. The reaction rate constant between FFA and ^1^O_2_ was carried out in triplet analysis.

#### 2.3.3. Determination of ^1^O_2_ Chemical Quenching Rate Constant

The ^1^O_2_ chemical quenching rates (*k_r_*) of antioxidants were determined by comparing their disappearance rates to that of FFA in methanol, using the known *k_r_^FFA^* value for FFA [31]. The sample solutions were prepared by dissolving FFA (0.5 × 10^−3^ M) and MB (6.25 × 10^−5^ M), and antioxidants (0.5 × 10^−3^ M) and MB (6.25 × 10^−5^ M) in methanol. Then, 10 mL of each sample solution was absorbed and added to a clean and transparent glass vial, and sealed with a screw cap. The prepared sample vials were transferred into an LED illumination box and exposed to 2000 lux of LED light for 25 min (sample contained FFA) or 50 min (sample contained antioxidant). At predetermined intervals, aliquots of sample solutions were taken out, and the residual FFA or antioxidants in the solutions were quantified by HPLC analysis; thus, the apparent reaction rates of FFA and antioxidants during light exposure could be calculated. With the known *k_r_^FFA^* value of FFA, the ^1^O_2_ chemical quenching rate constants of antioxidants were determined by measuring their relative oxidation rates to that of FFA in the same solution.

#### 2.3.4. Determination of FFA Contents by High-Performance Liquid Chromatography

The content of FFA was determined by using an Agilent Infinity 1260 HPLC (Agilent Technologies, Santa Clara, CA, USA) equipped with a UV–vis detector set at 219 nm for the detection of FFA [30]. FFA was separated using a Shim-Pack SB-C18 column (250 mm × 4.6 mm, 5 µm particle size, Shimadzu Scientific, Columbia, MD, USA) operating at 40 °C. The injection volume was 20 μL. An isocratic elution was applied: at first, a mixture of deionized water–acetonitrile (80:20, *v*/*v*) at a flow rate of 1.2 mL/min was maintained for 6.5 min to elute FFA. Then, the mobile phase was ramped to 100% acetonitrile in 20 s and maintained for 2.5 min to wash out any remaining components. Finally, the mobile phase was shifted back to the initial condition and held for 2 min to re-equilibrate the column. Agilent ChemStation (version B.04.03, Agilent Technologies, Santa Clara, CA, USA) was used for data collection and processing. FFA was identified by comparison of its retention time with that of the reference standard. The quantification of FFA was performed by using its external calibration function.

#### 2.3.5. Determination of the Antioxidants by High-Performance Liquid Chromatography

An Agilent Infinity 1260 HPLC equipped with a UV–vis detector and an automatic injector was used for the determination of antioxidants (α-tocopherol, BHA, TBHQ, EGCG, and quercetin). For α-tocopherol, the chromatographic condition was performed according to ISO 9936:2016 using a silica or diol-bonded silica column (250 mm × 4.6 mm, 5 μm), with n-hexane/isopropanol (99:1, *v*/*v*) as the mobile phase at a flow rate of 1.0 mL/min. Fluorescence detection was employed with an excitation wavelength of 295 nm and an emission wavelength of 330 nm [32]. For BHA and TBHQ, the chromatographic conditions were performed according to the AOAC Official Method 983.15 using a reversed-phase C18 column (100 mm × 2.1 mm, 2.6 μm). The mobile phase consisted of 0.1% formic acid in water (solvent A) and 0.1% formic acid in acetonitrile (solvent B). The flow rate was maintained at 0.3 mL/min, and the column temperature was controlled at 40 °C. Detection was achieved through electrospray ionization in positive mode (ESI^+^), with quantitative analysis by multiple reaction monitoring (MRM), and UV detection at 280 nm [33]. For EGCG, the chromatographic conditions were performed according to ISO/TC 34/SC 8 using a C18 analytical column (250 mm × 4.6 mm, 5 μm). The mobile phase consisted of solvent A (water containing acetic acid and disodium EDTA) and solvent B (acetonitrile) under gradient elution. The column temperature was maintained at 40 °C, and UV detection was performed at 278 nm [34]. For quercetin, reversed-phase HPLC was carried out following the method described by Wach et al. using a Luna C18(2) column (250 mm × 4.1 mm i.d., Phenomenex, Torrance, CA, USA). The mobile phase consisted of 25 mM of phosphate buffer (pH 2.5) and methanol operated in gradient elution mode. UV detection was performed at 370 nm as specified in the reference [35]. The identification of the antioxidants was achieved by comparing their retention time to those of the standard compounds. The quantification of the antioxidants was performed according to the peak ratio between each peak and that of the standard compound with known quantity.

### 2.4. Data Processing and Analysis

All experiments were carried out in three independent replicates, and the results were expressed as mean values ± standard deviation. Analysis of variance (ANOVA) with the Tukey HSD post hoc test was used to ascertain the statistical differences in total and chemical ^1^O_2_ quenching activities of the synthetic and natural antioxidants at α = 0.05 by using a SPSS 26.0 statistical analysis program (SPSS Inc., Chicago, IL, USA).

## 3. Results and Discussion

### 3.1. Determination of Total ^1^O_2_ Quenching Rate Constant (k_r_ + k_q_)

The total ^1^O_2_ quenching rate constants of five different antioxidants (α-tocopherol, BHA, TBHQ, EGCG, and quercetin) were determined via a steady-state kinetic equation. In the reaction, MB was used as a photosensitizer, and an LED was used as the light source to generate ^1^O_2_ via Type II photosensitive oxidation. Specifically, MB absorbs photons from LED light, typically in the red to visible range (around 660 nm wavelength). This promotes MB from its ground state (^1^MB) to an excited singlet state (^1^MB*). The ^1^MB* then undergoes intersystem crossing, forming the longer-lived triplet excited state (^3^MB*). The ^3^MB* then transfers its energy to ground-state molecular oxygen (^3^O_2_). This energy transfer excites ^3^O_2_ to ^1^O_2_, while the ^3^MB* returns to its ground state and can be reused in the cycle. When a solution containing a given quencher, MB, and FFA is exposed to radiation, the following reactions will happen:

(1) MB+O23 →light O21

(2) O21 →kd O23

(3) O21+FFA →krFFA FFA-O2

(4) O21+antioxidant→kr antioxidant-O2

(5) O21+antioxidant →kq antioxidant+O23

Note: Physical quenching of ^1^O_2_ by FFA is theoretically possible, but its contribution is negligible (*k*_phys_ ≪ *k*_chem_) and is therefore not considered in this work [29,36].

Where *k_d_* is the reciprocal of ^1^O_2_ lifetime in the solvent; *k_r_^FFA^* represents the chemical reaction rate constant of FFA with ^1^O_2_; *k_r_* represents the rate constant of chemical quenching of ^1^O_2_; *k_q_* represents the rate constant of physical quenching of ^1^O_2_; quencher-O_2_ is the quencher oxidation product.

In the reaction system without any antioxidant, ^1^O_2_ vanishes via two pathways: nonradiative decay (Reaction (2)) and deactivation by FFA (Reaction (3)). If the reaction system contains an antioxidant, ^1^O_2_ can be quenched through four distinct pathways, which are nonradiative decay (Reaction (2)), deactivation by FFA (Reaction (3)), ^1^O_2_ chemical quenching by the antioxidant (Reaction (4)), as well as ^1^O_2_ physical quenching by the antioxidant (Reaction (5)).

In our experiment, equal volumes of two solutions containing the same initial concentration of FFA (one with an antioxidant and the other without an antioxidant) were exposed to the same illuminating conditions. Although the antioxidants in the antioxidant addition group would also quench both the triplet excited state of MB (^3^MB*) and ^1^O_2_, under our experimental conditions, no more than about 4.3% of ^3^MB* was quenched by the antioxidants. Therefore, when different concentrations of antioxidants were added, the amount of ^1^O_2_ produced was approximately equal across different antioxidant concentrations [37,38]. Thus, the total ^1^O_2_ quenching rate constant of the antioxidant was calculated with Equation (6), which can be derived from steady-state kinetics [16,17]:

(6) $$S_{o}/S_{a}=1+\left[\left(k_{r}+k_{q}\right)/k_{d}\right][Q]$$

where *S_o_* represents the slope of the first-order curve for the vanishing of FFA (^1^O_2_ acceptor) in the absence of an antioxidant; *S_a_* represents the slope of the first-order curve for the vanishing of FFA when an antioxidant is present; and [Q] is the concentration of the antioxidant.

**Figure 1:**
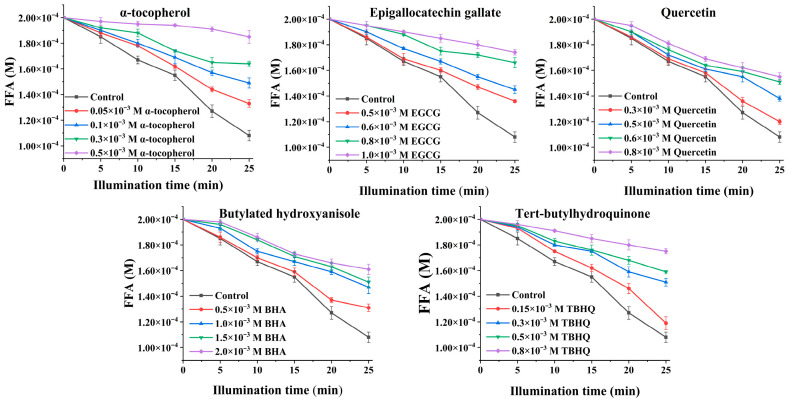
Effects of different concentrations of antioxidants on the FFA oxidation in methanol containing MB during LED light illumination (2000 Lux).

If plot *S_o_*/*S_a_* compared with [Q], a straight line would be obtained, whose slope is (*k_r_* + *k_q_*)/*k_d_*. By using Equation (6), the total ^1^O_2_ quenching rate constant (*k_r_* + *k_q_*) can be calculated. The ^1^O_2_ decay rate in methanol is 1.4 × 10^5^ s^−1^, as reported by Kim et al. [16] In a methanol system, the effects of antioxidants (α-tocopherol, BHA, TBHQ, EGCG, and quercetin) on FFA photooxidation (Type II pathway) were shown in Figure 1. The results showed that FFA photooxidation in methanol were inhibited by these antioxidants, and their inhibitory effects increased with the increase in antioxidant concentration. The photooxidation of FFA was shown in first-order kinetics.

**Figure 2:**
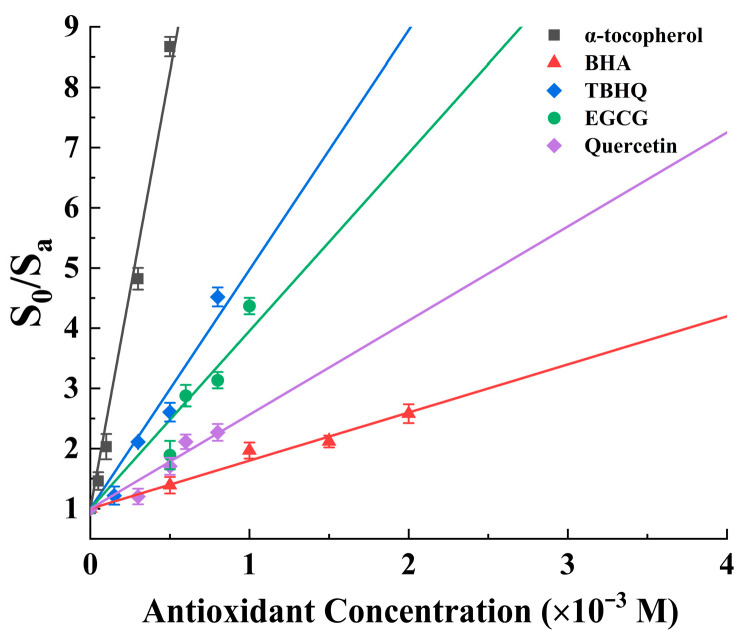
Plot of So/Sa against concentration of five antioxidants (α-tocopherol, BHA, TBHQ, EGCG, and quercetin).

The plots of *S_o_*/*S_a_* against [Q] for α-tocopherol, BHA, TBHQ, EGCG, and quercetin are shown in Figure 2. It can be seen that as the concentration of antioxidants in methanol increased, the values of S_o_/S_a_ also continuously increased (Figure 2), which means that the antioxidants had a dose-dependent protective effect on the oxidation of FFA induced by ^1^O_2_. The results revealed that the slope of α-tocopherol (Figure 2) was the steepest, followed by that of TBHQ, EGCG, quercetin, and BHA, which means that the protective activity of α-tocopherol against the FFA photooxidation was the highest. Compared with synthetic BHA, quercetin had better protective activity against the ^1^O_2_ oxidation of FFA.

With the known *k_d_* value (1.4 × 10^5^ s^−1^) and the slope of the plot shown in Figure 2, the total ^1^O_2_ quenching rate constant values of the five antioxidants can be calculated using Equation (6) and were 2.0 (±0.1) × 10^9^, 1.1 (±0.1) × 10^8^, 5.6 (±0.3) × 10^8^, 4.1 (±0.1) × 10^8^, and 2.2 (±0.1) × 10^8^ M^−1^s^−1^ for α-tocopherol, BHA, TBHQ, EGCG, and quercetin, respectively (Table 1). Our data revealed that the total ^1^O_2_ quenching rate of α-tocopherol was 18.1 times and 3.7 times greater than that of BHA and TBHQ, respectively, similar to reported data [16] in which the total ^1^O_2_ quenching rates of α-tocopherol were 10.5 times and 2.1 times greater than those of BHA and TBHQ, respectively. Our data also revealed that the total ^1^O_2_ quenching rate constants of EGCG and quercetin were within the same order of magnitude as that of TBHQ. As a natural antioxidant, α-tocopherol had approximately 4.9 times stronger ^1^O_2_ quenching activity than EGCG and approximately 9.3 times stronger ^1^O_2_ quenching activity than quercetin in our study. According to the data reported by Choi et al. [24], the total ^1^O_2_ quenching rate constant of α-tocopherol was approximately 2.9 times greater than that of EGCG, comparable to our results. Although the reaction system for the generation of ^1^O_2_ and the probe used for competitive reactions with the ^1^O_2_^−^ in previous reports were different from those in our study, the order of ^1^O_2_ quenching activity of the five examined antioxidants was the same.

After further analysis, we found that the absolute values of the total ^1^O_2_ quenching rate constants of the five antioxidants were different from those in previous reports. Lee and Jung [17] used MB as a photosensitizer and α-terpinene as a probe, generating ^1^O_2_ photochemically in methanol. In their experiment, the total ^1^O_2_ quenching rate constants of TBHQ and BHA were 1.99 × 10^8^ and 5.14 × 10^7^ M^−1^s^−1^, respectively. In contrast, our results yielded values of 5.6 × 10^8^ and 1.1 × 10^8^ M^−1^s^−1^ for TBHQ and BHA, representing 2.8-fold and 2.2-fold increases compared to their data [17]. Similarly, the total ^1^O_2_ quenching rate constant of EGCG (4.1 × 10^8^ M^−1^s^−1^) determined in our study is also higher than the value (1.31 × 10^8^ M^−1^s^−1^) reported by Choi and Jung [24], who used MB as a photosensitizer and α-terpinene as a probe, with ^1^O_2_ generated photochemically in methanol. In another study, Kim et al. [16] used DPBF as a probe, and ^1^O_2_ was produced by the dark chemical reaction of NaMoO_4_ and H_2_O_2_ in a methanol system. By this method, the total ^1^O_2_ quenching rate constants of BHA, α-tocopherol, and TBHQ were determined to be 3.37 × 10^7^, 3.54 × 10^8^, and 1.67 × 10^8^ M^−1^s^−1^, respectively, which were only 30.1%, 17.4%, and 30.0% of the values obtained in our study. Beyond methanol systems, the total ^1^O_2_ quenching rate constants of EGCG and quercetin have also been evaluated in ethanol solutions. When DPBF was used as a probe with ^1^O_2_ generated via thermal decomposition of endoperoxide, EGCG exhibited a total quenching rate constant of 1.47 × 10^8^ M^−1^s^−1^ [20]. In a separate study using rose bengal to generate ^1^O_2_, quercetin’s total quenching rate constant was determined as 1.9 × 10^6^ M^−1^s^−1^ via near-infrared fluorescence lifetime measurements [18]. Notably, our results for EGCG and quercetin showed 2.8-fold and 115.3-fold enhancements compared to these earlier studies.

The reasons for the differences in the total ^1^O_2_ quenching rate constants of BHA, TBHQ, EGCG, α-tocopherol, and quercetin between our data and previously reported values may be due to the following: (1) Poor solubility of the probe in methanol. In the reaction system, the probe and antioxidant compete for ^1^O_2_. To maintain a steady state—where the rate of ^1^O_2_ generation equals its disappearance (including self-quenching, quenching by the probe, and quenching by the antioxidant)—both components must remain within a reasonable concentration range. When the antioxidant concentration increases, the probe’s disappearance rate decreases to preserve this equilibrium. In previous reports [17], α-terpinene was used as a probe in methanol-based competitive systems. However, α-terpinene exhibits poor solubility in methanol, placing it at a disadvantage when competing with antioxidants for ^1^O_2_. Consequently, a larger proportion of the antioxidant is consumed during the reaction, leading to an underestimation of the total ^1^O_2_ quenching rate constant (derived from plotting *S*_0_*/S_a_* vs. [Q]) for the antioxidants compared to that obtained in our study. In contrast, our study employed FFA as the probe. FFA is highly soluble in methanol and exhibits specificity and high efficiency for ^1^O_2_ reactions. This allows FFA to compete effectively with antioxidants in a dose-dependent manner. Thus, the slope observed in *S*_0_*/S_a_* vs. [Q] plots in our study is steeper, reflecting that the total ^1^O_2_ quenching rate constant of the antioxidants exceeds previously reported data. (2) Lack of probe specificity for ^1^O_2_. Earlier work [16,23] utilized DPBF as a probe in competitive systems. However, DPBF is not specific to ^1^O_2_ and reacts with other reactive oxygen species (e.g., superoxide anions). To protect DPBF from nonspecific oxidation, a greater amount of antioxidant is consumed compared to systems using a ^1^O_2_-specific probe. This artifact could reduce the calculated total ^1^O_2_ quenching rate constant. By using FFA—a probe with exclusive specificity for ^1^O_2_ and a high reaction rate constant—we eliminated interference from nonspecific reactions. This methodological refinement resulted in higher total ^1^O_2_ quenching rate constants for BHA, α-tocopherol, and TBHQ in our study compared to prior reports [16,23]. (3) The solvent effect plays a critical role in modulating reaction kinetics. Notably, methanol exhibits significantly higher polarity than ethanol. In this study, the total ^1^O_2_ quenching rate constant of quercetin was determined in methanol, whereas the previous study [18] utilized ethanol. As solvent polarity increases, the Gibbs free energy of the charge transfer state decreases, leading to an enlarged standard free energy difference (|Δ*G*^0^|). According to the Marcus equation [39], this reduction in activation energy (Δ‡*G*) enhances the electron transfer rate constant (*k*_et_), thereby driving the observed increase in the total singlet oxygen quenching rate constants. This mechanistic interpretation explains why the total ^1^O_2_ quenching rate constant of quercetin reported in this study is substantially higher than that documented in earlier literature. The influence of solvent polarity on the total singlet oxygen quenching rate constant of antioxidants can be further demonstrated through comparative studies of α-tocopherol in different solvents. For instance, Mukai et al. [40,41] reported that the total quenching rate constant of α-tocopherol determined in ethanol was 2.06 × 10^8^ M^−1^s^−1^, whereas in an ethanol/chloroform/D_2_O (50:50:1) mixture, the value was 1.35 × 10^8^ M^−1^s^−1^ [42]. Comparative analysis reveals a positive correlation between solvent polarity and the total quenching rate constant: higher-polarity solvents (e.g., ethanol, polarity index 5.2) exhibit a higher value compared to lower-polarity solvents (e.g., the ethanol/chloroform/D_2_O mixture, polarity index 4.7–4.8). Specifically, the value in ethanol is 1.5 times that in the mixed solvent (2.06 × 10^8^ vs. 1.35 × 10^8^ M^−1^s^−1^).

### 3.2. Determination of ^1^O_2_ Reaction Rate Constant of FFA

**Figure 3:**
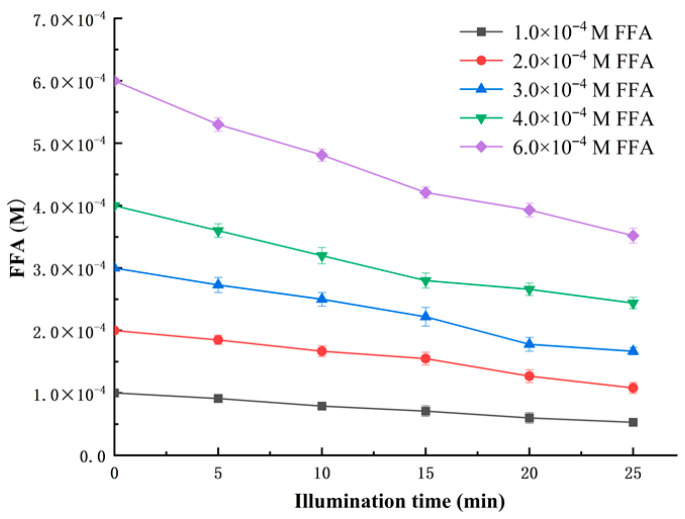
Plot of FFA oxidation at different concentrations in methanol containing MB under LED light illumination (2000 Lux).

The ^1^O_2_ oxidation rate constant of FFA was calculated using Equation (7).

(7) $${\left\{-d[\mathrm{FFA}]/dt\right\}}^{-1}=K^{-1}\left\{1+\left(k_{d}/k_{r}^{FF4}[\mathrm{FFA}]\right)\right\}$$

where *−d* [FFA]/*dt* is the instantaneous disappearance rate of FFA (also known as the apparent disappearance rate); K^−1^ is reciprocal of the rate of ^1^O_2_ generation; *k_d_* represents the decay rate of ^1^O_2_ in the solvent; and *k_r_^FFA^* is the chemical reaction rate constant of FFA reacted with ^1^O_2_. By plotting {−*d*[FFA]/*dt*}^−1^ against [FFA]^−1^, a straight line would be obtained whose intercept is K^−1^ and slope is *β* × K^−1^. Then, a value of *β* would be obtained by dividing the slope by the intercept, that is, *β* = *k_d_*/*k_r_^FFA^*. The ^1^O_2_ decay rate (*k_d_*) in methanol is 1.4 × 10^5^ s^−1^, as reported by Kim et al. [16]. Figure 3 shows the photooxidation of FFA in methanol by using MB as a photosensitizer and LED as a light source. FFA oxidation followed first-order kinetics.

**Figure 4:**
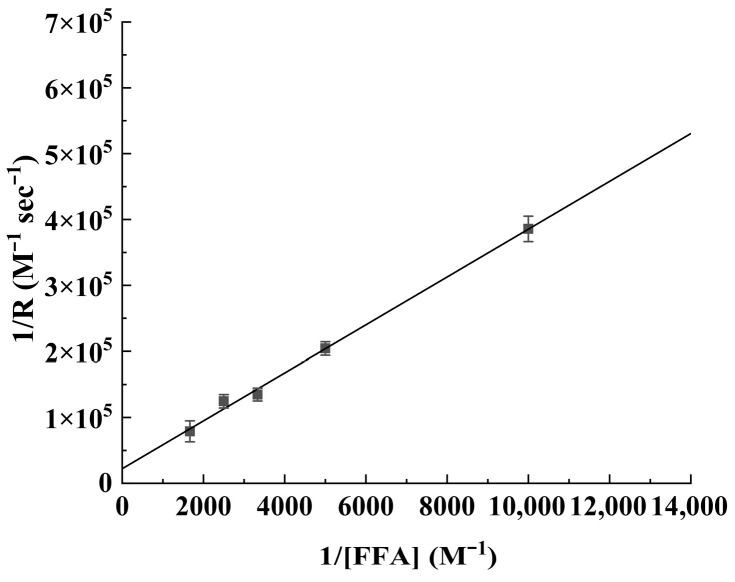
Plot of 1/R against 1/C for the oxidation of FFA.

Figure 4 shows the plot of 1/R (reciprocal of apparent rate of FFA disappearance, obtained from (Figure 3)) against 1/C (reciprocal of FFA concentration). According to the plot (Figure 4), the value of *β* obtained from three replicates of independent experimental analysis was 0.002210. With the known values of *k_d_* and *β*, *k_r_^FFA^* can be calculated: *k_r_^FFA^* = *k_d_*/*β* = 1.4 × 10^5^/0.002210 = 6.33 × 10^7^ M^−1^s^−1^.

### 3.3. Determination of ^1^O_2_ Chemical Quenching Rate Constant (k_r_) and Physical Quenching Rate Constant (k_q_) of Antioxidants

The ^1^O_2_ chemical quenching rate (*k_r_*) of the antioxidants in methanol was determined according to the relative apparent photooxidation rate of the antioxidants compared with the photooxidation rate of FFA, irradiated under the same conditions.

(8) *k_r_^antioxidant^*/*k_r_^FFA^* = {−*d* [antioxidant]/*dt*}/{−*d* [FFA]/*dt*}

where *k_r_^FFA^* represents the chemical reaction rate constant of FFA with ^1^O_2_; *k_r_^antioxidant^* represents the chemical reaction rate constant of the antioxidant with ^1^O_2_; −*d*[FFA]/*dt* represents the apparent rate of FFA disappearance; and −*d*[antioxidant]/*dt* is the apparent rate of antioxidant disappearance.

**Figure 5:**
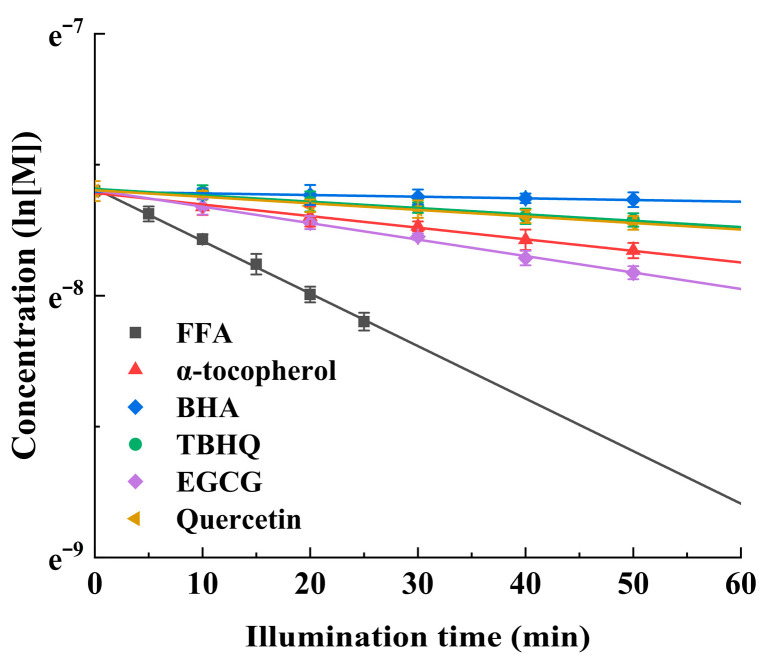
Relative ^1^O_2_ reaction rates of α-tocopherol, BHA, TBHQ, EGCG, and quercetin in methanol solution as compared with FFA. “ln” denotes the natural logarithm and “[M]” represents the concentration of the substance (FFA or an antioxidant) at time t.

The disappearance rates of α-tocopherol, BHA, TBHQ, EGCG, and quercetin compared with FFA in a methanol solution containing MB during illumination under the same conditions are shown in Figure 5, which shows that the disappearance of the antioxidants followed first-order kinetics. The apparent rate constants (*k*) of FFA, α-tocopherol, BHA, TBHQ, EGCG, and quercetin calculated by using the first-order kinetic equation were −2.01 × 10^−2^ min^−1^, −4.4 × 10^−3^ min^−1^, −6.35 × 10^−4^ min^−1^, −2.43 × 10^−3^ min^−1^, −6.29 × 10^−3^ min^−1^, and −2.50 × 10^−3^ min^−1^, respectively. After that, the relative reaction rates for α-tocopherol, BHA, TBHQ, EGCG, and quercetin compared with that of FFA were calculated to be 0.219, 0.032, 0.121, 0.313, and 0.124, respectively. The results showed that EGCG was most reactive to ^1^O_2_, followed by α-tocopherol, quercetin, TBHQ, and BHA, in decreasing order. With Equation (8), the ^1^O_2_ chemical quenching rate constants for α-tocopherol, BHA, TBHQ, EGCG, and quercetin can be calculated: *k_r_^α-tocopherol^* = 0.219 × 6.33 × 10^7^ = 1.4 × 10^7^ M^−1^s^−1^, *k_r_^BHA^* = 0.032 × 6.33 × 10^7^ = 2.0 × 10^6^ M^−1^s^−1^, *k_r_^TBHQ^* = 0.121 × 6.33 × 10^7^ = 7.7 × 10^6^ M^−1^s^−1^, *k_r_^EGCG^* = 0.313 × 6.33 × 10^7^ = 2.0 × 10^7^ M^−1^s^−1^, and *k_r_^quercetin^* = 0.124 × 6.33 × 10^7^ = 7.9 × 10^6^ M^−1^s^−1^ (Table 1). To the best of our knowledge, no previously reported data are available for the ^1^O_2_ chemical quenching rate constant of quercetin in methanol. The *k_r_* values of BHA and TBHQ, which are commonly used in synthetic antioxidants, greatly differ. The *k_r_* values of BHA and TBHQ were determined by Lee and Jung [17] as 3.90 × 10^5^ and 2.93 × 10^6^ M^−1^s^−1^, respectively, with the latter being approximately 7.5 times higher than the former. In our study, the *k_r_* value of TBHQ was approximately 3.8 times higher than that of BHA. In addition, the *k_r_* values of synthetic antioxidants and natural antioxidants were very different. The *k_r_* values of α-tocopherol and EGCG were significantly greater than those of BHA and TBHQ. The *k_r_* value of BHA was only approximately 1/9.8 of that of EGCG, which means that BHA would remain as an intact active form for approximately 9.8 times longer than EGCG in the presence of ^1^O_2_. For the three natural antioxidants, the *k_r_* value of quercetin was the smallest compared with those of α-tocopherol and EGCG, indicating that the duration of quercetin activity was approximately 2.52 and 1.67 times longer than that of EGCG and α-tocopherol, respectively. The percentages of chemical quenching in total ^1^O_2_ quenching, *k_r_*/(*k_r_* + *k_q_*), were 0.7%, 1.8%, 1.4%, 4.8%, and 3.6% for α-tocopherol, BHA, TBHQ, EGCG, and quercetin, respectively (Table 1). The data (*k_r_* + *k_q_*)/*k_r_* indicates that one α-tocopherol molecule can deactivate approximately 146 molecules of ^1^O_2_, whereas single molecules of EGCG, quercetin, TBHQ, or BHA deactivate approximately 21, 28, 72, or 55 molecules of ^1^O_2_, respectively, before being oxidized. That is, α-tocopherol, BHA, TBHQ, EGCG, and quercetin quenched ^1^O_2_ almost exclusively via a physical quenching mechanism. Approximately 99.3%, 98.2%, 98.6%, 95.2%, and 96.4% of the ^1^O_2_ quenching by α-tocopherol, BHA, TBHQ, EGCG, and quercetin occurred through their physical quenching mechanism. However, a previous study [23] reported a lower proportion of physical quenching for α-tocopherol (92.6%) compared to our result (99.3%). This discrepancy may arise from the low specificity of DPBF in competitive reaction systems [43] and from the experimental conditions (e.g., the method of ^1^O_2_ generation, the sensitivity of the detection methods for α-tocopherol and DPBF), both of which may overestimate the chemical quenching reaction. To evaluate the antioxidant capability of a ^1^O_2_ scavenger, it is essential to determine its ^1^O_2_ chemical quenching rate constant and total ^1^O_2_ quenching rate constant. If the ratio of the chemical quenching rate constant to the total quenching rate constant of an antioxidant is high, the antioxidant will degrade rapidly due to excessive chemical consumption, resulting in limited operational lifespan during its protective role against ^1^O_2_-mediated oxidation of bioactive components or vegetable oils. Our results show that the five antioxidants could protect vegetable oil from ^1^O_2_ oxidation without significant degradation during the prolonged period.

## 4. Conclusions

In this study, methanol-soluble FFA was used as a probe for competitive reactions with an antioxidant in a methanol system. Owing to FFA’s solubility in methanol, its specificity for ^1^O_2_, and its high reaction rate constant with ^1^O_2_, the total ^1^O_2_ quenching rate constants of α-tocopherol, BHA, TBHQ, EGCG, and quercetin determined under these conditions were higher than previously reported. The values obtained in our study are 2.0 (±0.1) × 10^9^, 1.1 (±0.1) × 10^8^, 5.6 (±0.3) × 10^8^, 4.1 (±0.1) × 10^8^, and 2.2 (±0.1) × 10^8^ M^−1^s^−1^, respectively. According to the *k_r_* value of FFA determined first, the *k_r_* values of the five antioxidants were calculated by measuring their relative reaction rates with ^1^O_2_ to that of FFA under the same conditions. The *k_r_* values of α-tocopherol, BHA, TBHQ, EGCG, and quercetin were 1.4 × 10^7^, 2.0 × 10^6^, 7.7 × 10^6^, 2.0 × 10^7^, and 7.9 × 10^6^ M^−1^s^−1^, respectively. This is the first report on the ^1^O_2_ chemical quenching rate constant of quercetin in methanol. The total ^1^O_2_ quenching rate constant of α-tocopherol was greater than that of quercetin, but the antioxidant life of α-tocopherol was shorter than that of quercetin. The ratios of chemical quenching over total ^1^O_2_ quenching, *k_r_*/(*k_r_* + *k_q_*), were 0.7%, 1.8%, 1.4%, 4.8%, and 3.6% for α-tocopherol, BHA, TBHQ, EGCG, and quercetin, respectively, indicating that the five antioxidants quenched ^1^O_2_ almost exclusively by means of physical quenching and could protect vegetable oil from ^1^O_2_ oxidation without significant degradation during storage.

## Figures and Tables

**Table 1:** Total ^1^O_2_ quenching rate constant (*k_r_* + *k_q_*), ^1^O_2_ chemical quenching rate constant (*k_r_*), ratio of chemical quenching rate over total quenching rate (*k_r_*/*k*_r_ + *k_q_*), and ratio of physical quenching rate over total quenching rate (1 − *k_r_*/*k_r_* + *k_q_*).

Antioxidant	Total Quenching Constant k_r_ + k_q_ (M^−1^s^−1^)	Chemical Quenching Constant k_r_ (M^−1^s^−1^)	Chemical Quenching Ratio k_r_/k_r_ + k_q_	Physical Quenching Ratio 1 − k_r_/k_r_ + k_q_
α-tocopherol	2.0 (±0.1) × 10^9^	1.4 × 10^7^	0.7%	99.3%
BHA	1.1 (±0.1) × 10^8^	2.0 × 10^6^	1.8%	98.2%
TBHQ	5.6 (±0.3) × 10^8^	7.7 × 10^6^	1.4%	98.6%
EGCG	4.1 (±0.1) × 10^8^	2.0 × 10^7^	4.8%	95.2%
Quercetin	2.2 (±0.1) × 10^8^	7.9 × 10^6^	3.6%	96.4%

## Data Availability

The original contributions presented in this study are included in the article; further inquiries can be directed to the corresponding author.

## Abbreviations

The following abbreviations are used in this manuscript:

FFA	furfuryl alcohol
^1^O_2_	singlet oxygen
BHA	butylated hydroxyanisole
TBHQ	tert-butylhydroquinone
EGCG	epigallocatechin gallate
BHT	tert-di-butylhydroxytoluene
MB	methylene blue
